# Supplementary figures and images for: VLA-4 suppression by senescence signals regulates meningeal immunity and leptomeningeal metastasis
Source: eLife. 2022 Dec 9;11:e83272. doi: 10.7554/eLife.83272 (PMC9803356; doi:10.7554/eLife.83272)

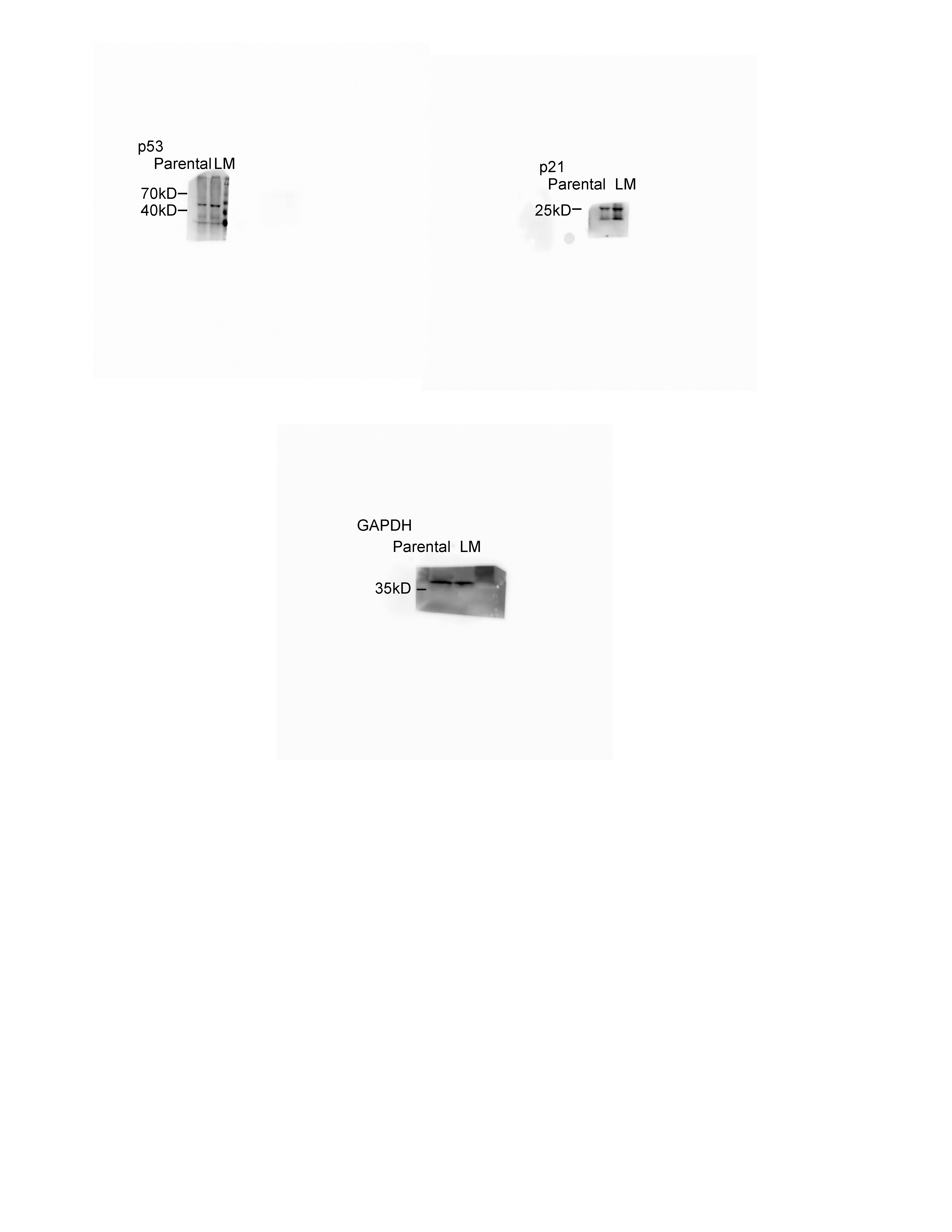

Supplement: Figure 4—source data 1. [file elife-83272-fig4-data1.zip › Figure 4í¬source data 1. Uncropped blots in Figure 4G/Figure 4í¬source data 1. Uncropped blots with the relevant bands clearly labelled in Figure 4G.tif]

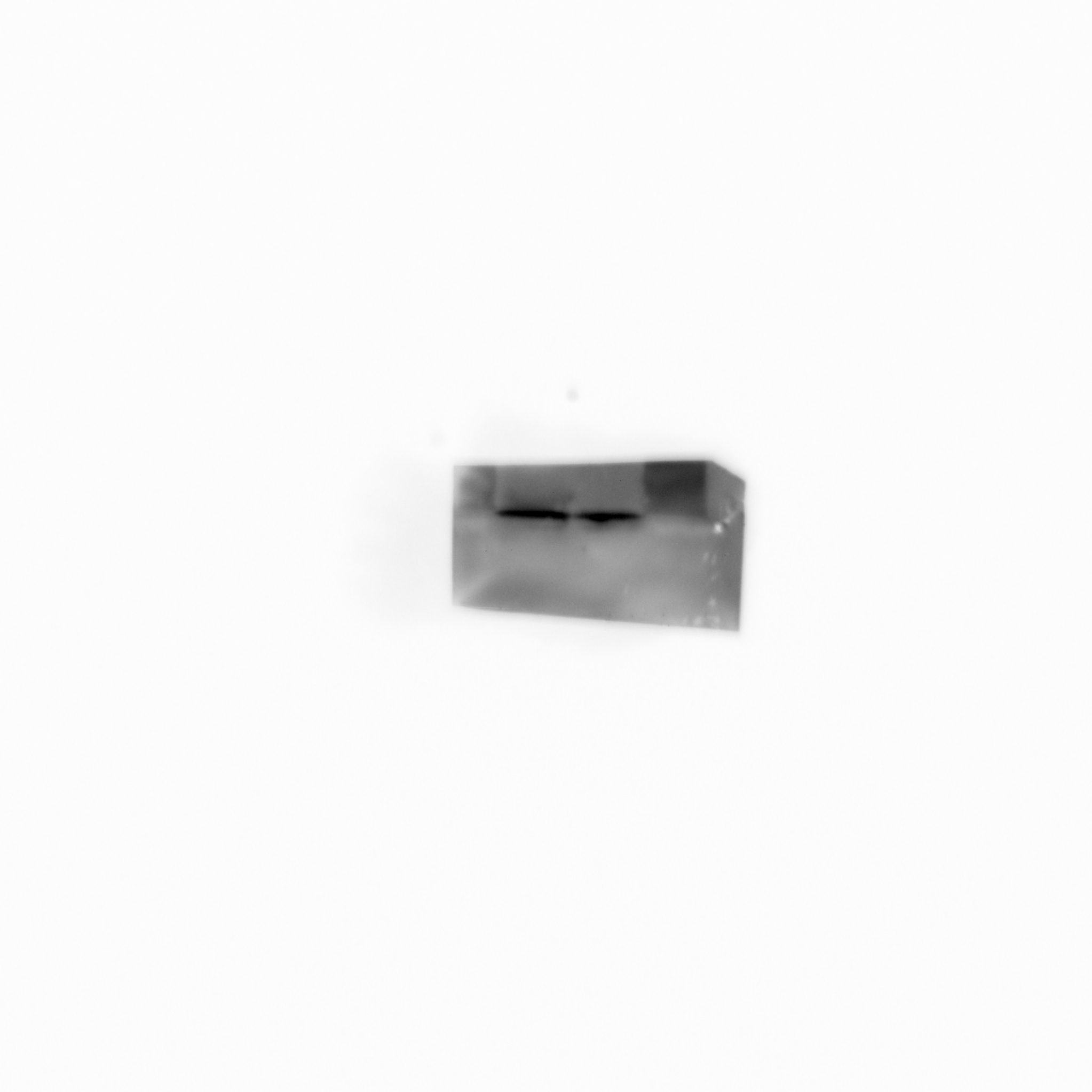

Supplement: Figure 4—source data 1. [file elife-83272-fig4-data1.zip › Figure 4í¬source data 1. Uncropped blots in Figure 4G/Figure 4í¬source data 2. Uncropped blots in Figure 4G gapdh.tif]

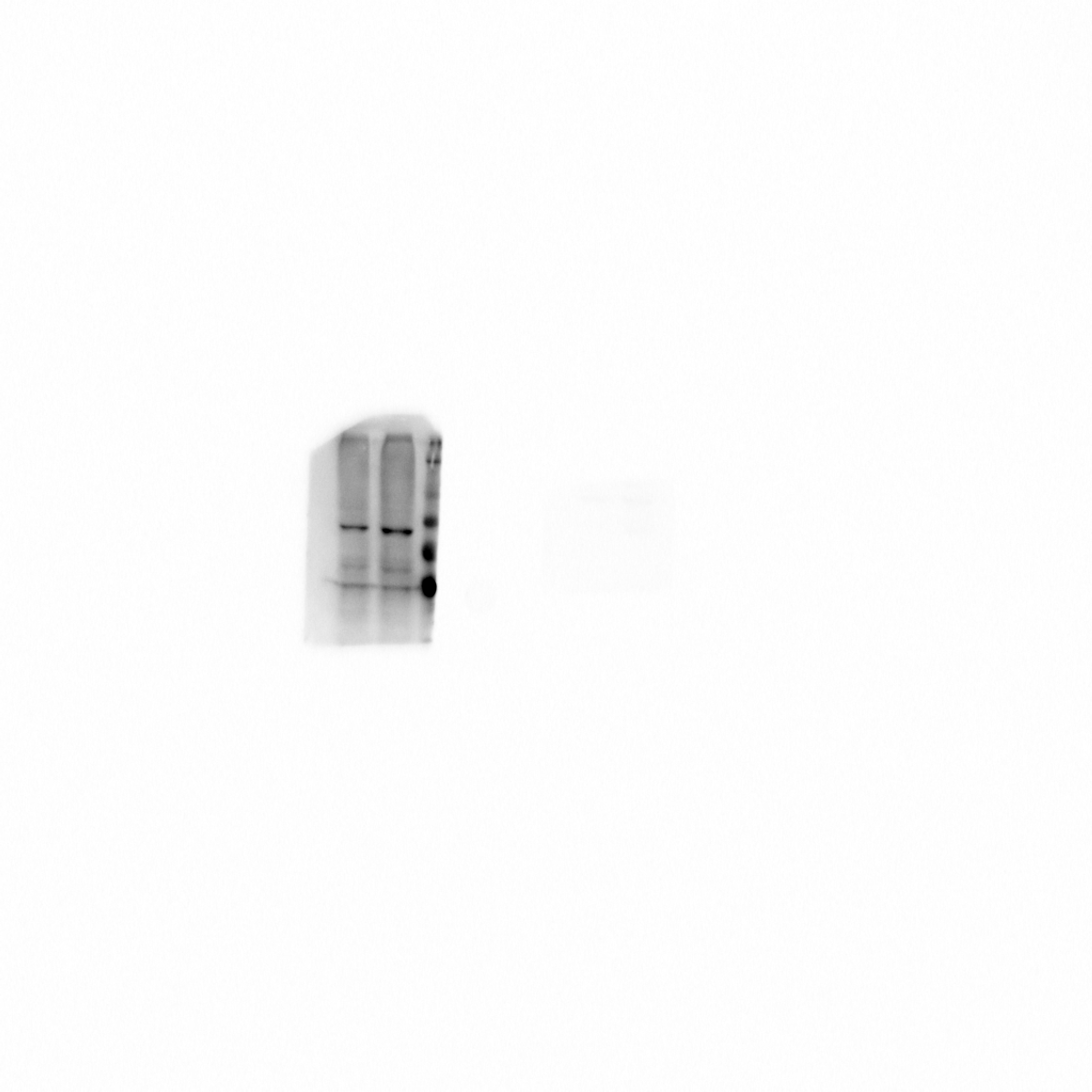

Supplement: Figure 4—source data 1. [file elife-83272-fig4-data1.zip › Figure 4í¬source data 1. Uncropped blots in Figure 4G/Figure 4í¬source data 2. Uncropped blots in Figure 4G p53.tif]

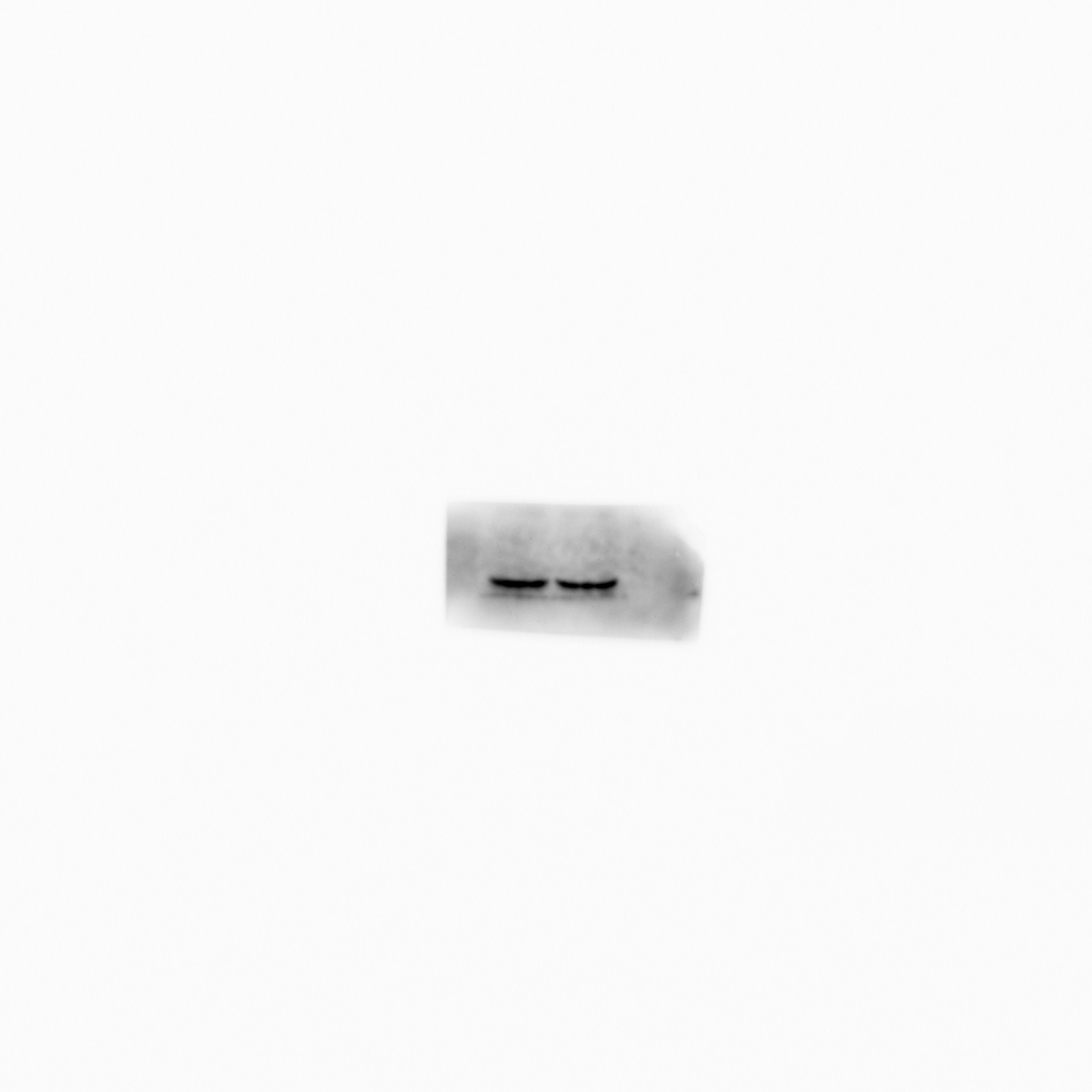

Supplement: Figure 4—figure supplement 1—source data 1. [file elife-83272-fig4-figsupp1-data1.zip › Figure supplement 4í¬source data 1. Uncropped blots in Figure supplement 4I/Figure supplement 4í¬source data 1. Uncropped blots in Figure supplement 4I gapdh.tif]

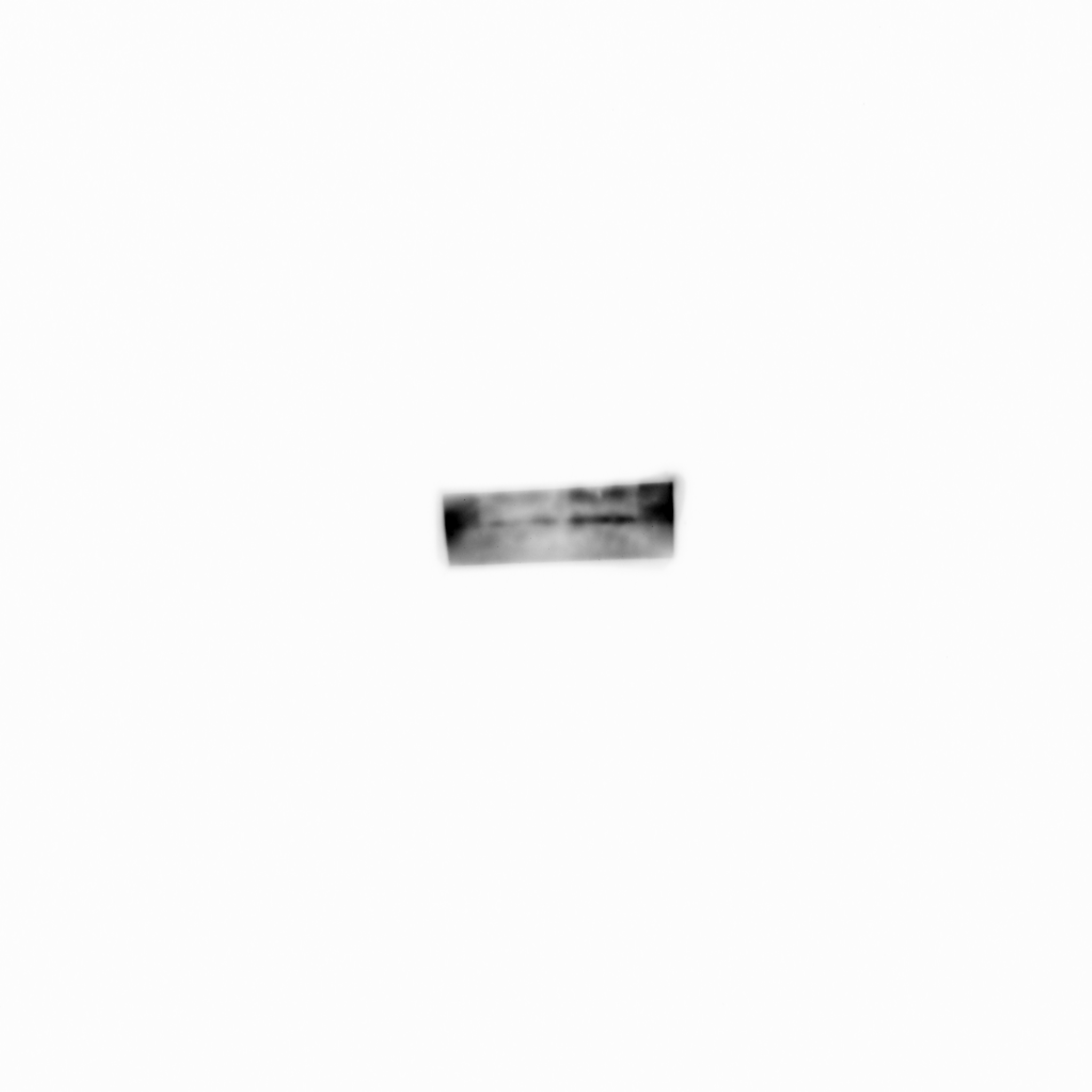

Supplement: Figure 4—figure supplement 1—source data 1. [file elife-83272-fig4-figsupp1-data1.zip › Figure supplement 4í¬source data 1. Uncropped blots in Figure supplement 4I/Figure supplement 4í¬source data 1. Uncropped blots in Figure supplement 4I p21.tif]

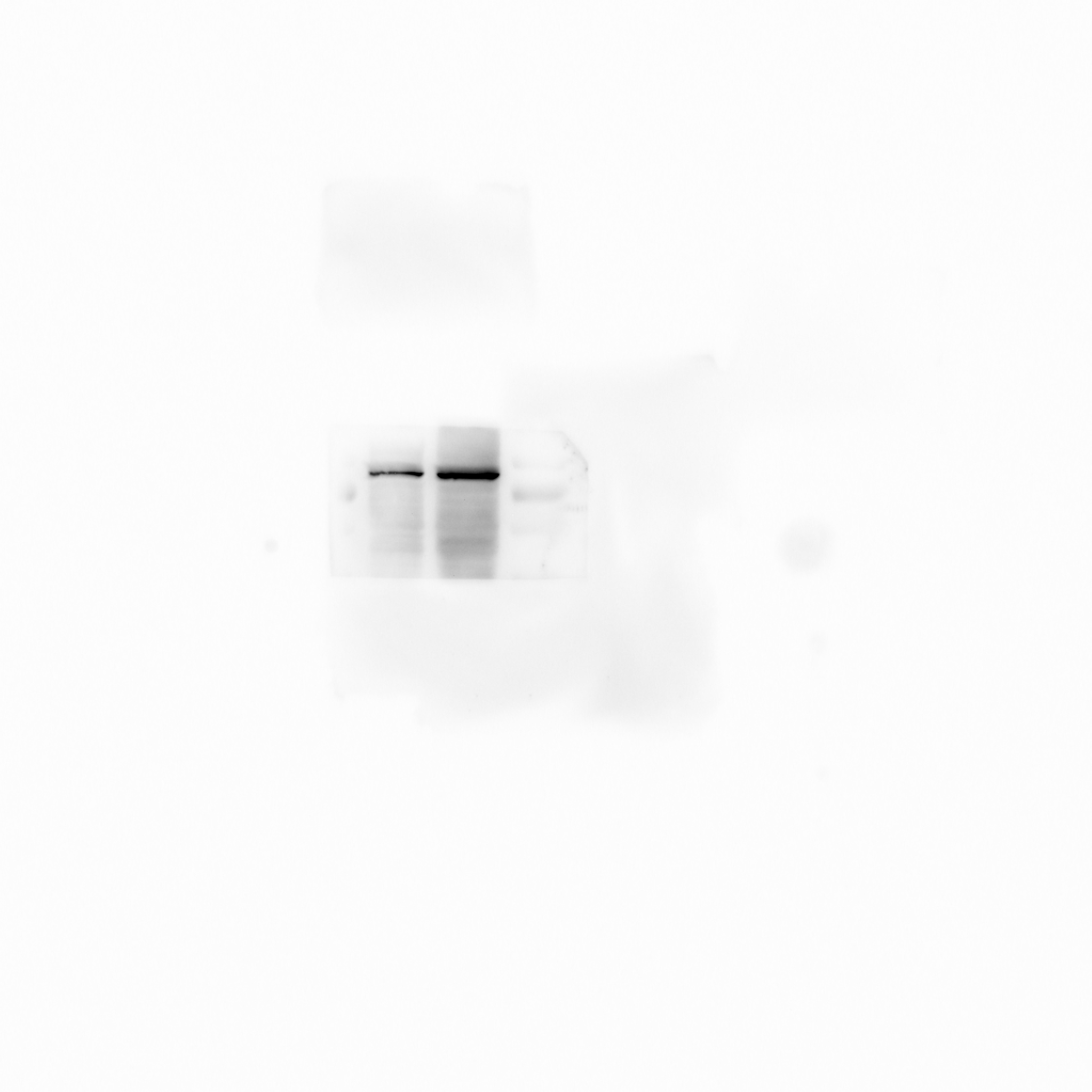

Supplement: Figure 4—figure supplement 1—source data 1. [file elife-83272-fig4-figsupp1-data1.zip › Figure supplement 4í¬source data 1. Uncropped blots in Figure supplement 4I/Figure supplement 4í¬source data 1. Uncropped blots in Figure supplement 4I p53.tif]

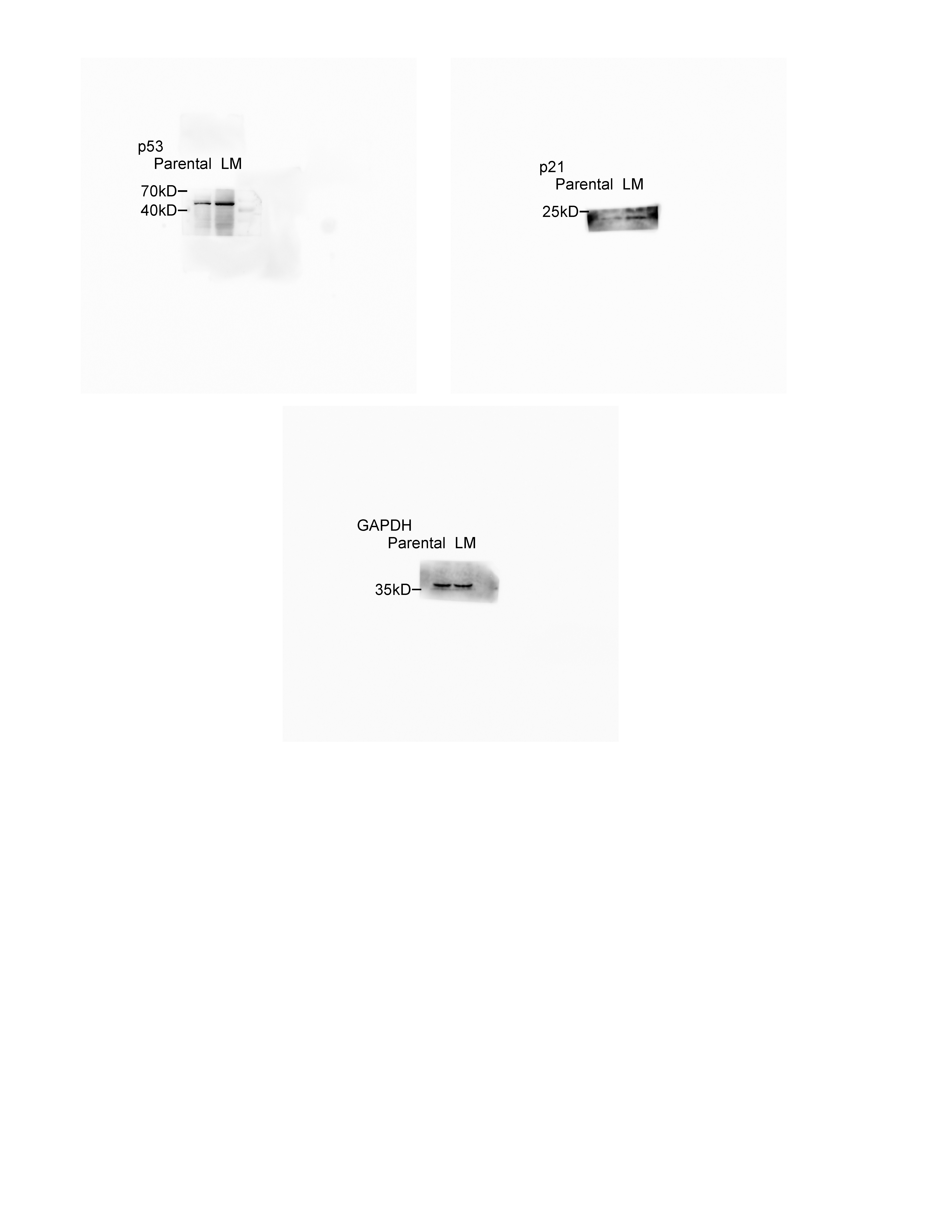

Supplement: Figure 4—figure supplement 1—source data 1. [file elife-83272-fig4-figsupp1-data1.zip › Figure supplement 4í¬source data 1. Uncropped blots in Figure supplement 4I/Figure supplement 4í¬source data 1. Uncropped blots with the relevant bands clearly labelled in Figure supplement 4I.tif]
